# Highlights and recent developments in airway diseases in EAACI journals (2017)

**DOI:** 10.1186/s13601-018-0238-3

**Published:** 2018-11-27

**Authors:** J. Bousquet, C. A. Akdis, C. Grattan, P. A. Eigenmann, K. Hoffmann-Sommergruber, P. W. Hellings, I. Agache

**Affiliations:** 1 MACVIA-France, Fondation partenariale FMC VIA-LR, Montpellier, France; 2 INSERM U 1168, VIMA: Ageing and Chronic Diseases Epidemiological and Public Health Approaches, Villejuif, France; 3 UMR-S 1168, Université Versailles St-Quentin-en-Yvelines, Montigny le Bretonneux, France; 4 Euforea, Brussels, Belgium; 5 Swiss Institute of Allergy and Asthma Research (SIAF), University Zurich, Davos, Switzerland; 6 St John’s Institute of Dermatology, Guy’s Hospital, London, UK; 7 Pediatric Allergy Unit, University Hospitals of Geneva, Geneva, Switzerland; 8 Department of Pathophysiology and Allergy Research, Medical University of Vienna, Vienna, Austria; 9 Laboratory of Clinical Immunology, Department of Microbiology and Immunology, KU Leuven, Louvain, Belgium; 10 Transylvania University Brasov, Brasov, Romania; 11 CHRU Arnaud de Villeneuve, 371 Avenue du Doyen Gaston Giraud, 34295 Montpellier Cedex 5, France

**Keywords:** Asthma, Rhinitis, EAACI

## Abstract

The European Academy of Allergy and Clinical Immunology (EAACI) owns three journals: Allergy, Pediatric Allergy and Immunology and Clinical and Translational Allergy. One of the major goals of EAACI is to support health promotion in which prevention of allergy and asthma plays a critical role and to disseminate the knowledge of allergy to all stakeholders including the EAACI junior members. There was substantial progress in 2017 in the identification of basic mechanisms of allergic and respiratory disease and the translation of these mechanisms into clinics. Better understanding of molecular and cellular mechanisms, efforts for the development of biomarkers for disease prediction, novel prevention and intervention studies, elucidation of mechanisms of multimorbidies, entrance of new drugs in the clinics as well as recently completed phase three clinical studies and publication of a large number of allergen immunotherapy studies and metaanalyses have been the highlights of the last year.

## Introduction

The European Academy of Allergy and Clinical Immunology (EAACI) has three official journals: Allergy, Pediatric Allergy and Immunology and Clinical and Translational Allergy. One of the major goals of EAACI is to support health promotion in which prevention and control of allergy plays a critical role and to disseminate the knowledge of allergy to all stakeholders including the EAACI junior members.

The EAACI journals have reported on the prediction and primary and secondary prevention of allergic diseases and asthma, and food allergy in 2016 [1, 2]. This paper summarises the achievements of 2017 in asthma and rhinitis. Completed and published EAACI task forces are listed in Table 1.

**Table 1:** List of task force articles published

Manuscript title	References
Overview of systematic reviews in allergy epidemiology	[15]
Allergy in severe asthma	[80]
The burden of nonadherence among adults with asthma: a role for shared decision-making	[48]
Defining pollen exposure times for clinical trials of allergen immunotherapy for pollen-induced rhinoconjunctivitis—an EAACI position paper	[81]
AllergoOncology—the impact of allergy in oncology: EAACI position paper	[82]
Allergen exposure chambers: harmonizing current concepts and projecting the needs for the future—an EAACI Position Paper	[26]
Biomarkers for monitoring clinical efficacy of allergen immunotherapy for allergic rhinoconjunctivitis and allergic asthma: an EAACI Position Paper	[83]
Positioning the principles of precision medicine in care pathways for allergic rhinitis and chronic rhinosinusitis—A EUFOREA-ARIA-EPOS-AIRWAYS ICP statement	[46]
Diagnostic tools in ocular allergy	[84]
Work productivity in rhinitis using cell phones: the MASK pilot study	[75]
Non-allergic rhinitis: position paper of the European Academy of Allergy and Clinical Immunology	[85]

## Prediction and prevention

The preventive role of nutrition in allergy and asthma is still a matter of debate and studies are ongoing. In the PACMA study (NL), breastfeeding was associated with a decreased risk of childhood asthma exacerbations later in life [3]. A meta-analysis found that fish intake in infancy could reduce the risk of eczema and allergic rhinitis in children, whereas maternal fish intake during pregnancy does not affect any atopic outcome. The intake of fish per se in infancy, not specially n-3 LC-PUFAs, may have an allergy protective effect. High-quality and adequately powered RCTs are however needed for a final conclusion [4]. However, a balance between primary prevention of allergic disease and optimal infant growth and nutrition should always be considered [5].

Is there a march from early food sensitization to later childhood allergic airway disease? Two prospective birth cohort studies [the high-risk Melbourne Atopic Cohort Study (MACS) (n = 620) and the population-based LISAplus (n = 3094)] showed that food sensitization (with or without aeroallergen sensitization) in the first 2 years of life increased the risk of subsequent asthma and allergic rhinitis [6]. Life-course of atopy and allergy-related disease events are rarely studied in tropical sub-Saharan Africa. 2345 Ugandan children were followed from birth to 9 years. Allergen sensitization started early in childhood and increased with age. Eczema and wheeze were common in infancy and declined with age. Atopy was strongly associated with asthma, rhinitis and eczema among the few affected children. Nevertheless, the typical Atopic March did not occur [7].

The early prediction of allergic diseases is one of the most important question in pediatric allergy. The predictive value of serum sST2 in preschool wheezers was observed for development of asthma with high FeNO [8]. A prospective birth cohort study (Danish Allergy Research Center cohort) identified early childhood risk factors for allergic and non-allergic rhinoconjunctivitis in adolescence [9]. Eczema which commences in early infancy and persists into toddler years is strongly associated with asthma, and to a lesser extent hay fever, in high-risk children. If these associations are causal, prevention of early-life eczema might reduce the risk of respiratory allergy [10]. Indoor fungal diversity in primary schools may differently influence allergic sensitization and asthma in children [11].

Long-term perinatal probiotic intervention was safe but efficacy was difficult to be confirmed in four randomized, double-blind, placebo-controlled trials [12]. In Uganda, treating helminths during pregnancy and early childhood in the absence of change in other exposures, is unlikely to increase the risk of atopic diseases later in childhood [13].

## Epidemiology

Birth decade affects the sensitization pattern and asthma risk were studied in Finnish adult population population-based case–control data (N = 456). Sensitization to more than one allergen type and the number of positive SPT reactions associated with younger age and asthma. Atopic subjects aged 65 and above were characterized by sensitization to only one to two allergens [14].

The state of asthma epidemiology was assessed by the Task Force “Overview of Systematic Reviews in Allergy Epidemiology” of the EAACI Interest Group on Epidemiology that proposed an overview of systematic reviews and their quality [15]. The same Task Force published an overview of systematic reviews in allergy epidemiology [16].

## Multimorbidity

In allergic diseases, the term co-morbidity should be replaced by multimorbidity since the primary organ is unknown as indicated by a EAACI Task Force Report [17]. Several papers dealt with multimorbidity in the three journals. Allergen sensitization affects the change trend of prevalence of symptoms of rhinitis coexisting with wheeze among adolescents in Guangzhou City from 1994 to 2009 [18]. *Staphylococcus aureus* enterotoxin sensitization is associated with allergic poly-sensitization and allergic multimorbidity in adolescents [19]. The gender-shift in prevalence of allergic rhinitis and comorbid asthma from childhood to adulthood is more important in multimorbid disease [20].

There was no consistent evidence for an association of asthma or chronic rhinosinusitis with fruit and vegetable intake in this representative sample of European adults (Global Allergy and Asthma Network of Excellence (GA^2^LEN) Survey) [21].

In rhinitis and asthma, CHRODIS criteria were applied to the MASK (Mobile Airways Sentinel NetworK) that has been recognized as a Good Practice [22].

## Diagnosis

The diagnosis of allergy is changing with new in vitro tests and the refinement of older ones. Diagnosing allergic sensitizations in the third millennium is important and clinicians should know allergen molecule structures [23].

The high prevalence of food and respiratory sensitization supports the clinical guideline recommendation that allergies should be evaluated in all children with suspected asthma. The microarray platforms used found acceptable accuracy and provided refined IgE characterization in 47% of the patients compared to standard extract-based methods [24]. The Pasture Study Group compared skin prick tests and specific IgE in 10-year-old children. Skin prick test and specific IgE display moderate agreement, but have a similar Area Under the Curve for allergic diseases. At the cutoff value of 3 mm for skin prick test and 0.35 IU/mL for specific IgE, skin prick test T has a higher specificity for asthma and hay fever than specific IgE without difference for sensitivity [25].

Many allergen exposure chambers exist and a EAACI Position paper attempted to harmonize current concepts and projecting the needs for the future [26]. Peak nasal inspiratory flow was found to be as outcome for provocation studies in allergen exposure chambers in a GA^2^LEN study [27].

Airways basophils may be of relevant important in the diagnosis of asthma [28]. They are increased and activated in eosinophilic asthma [29]. Sputum basophils are increased in eosinophilic asthma compared with non-eosinophilic asthma phenotypes [30].

Some new markers may be useful in severe asthma including airway and peripheral urokinase plasminogen activator receptor [31] and CD48 on blood leukocytes [32]. Data from the large population-based cohort Swedish GA^2^LEN show that serum periostin relates to type-2 inflammation and lung function in asthma [33]. As MicroRNAs (miRNAs), that regulate gene expression at the post-transcriptional level, are altered in asthma, circulating miRNAs are attractive candidates for the identification of novel biomarkers. Distinct plasma miRNAs are differentially regulated in human allergic asthma and were associated with clinical characteristics of patients. miRNA levels in plasma might have future potential to subphenotype patients with asthma [34].

## Mechanisms

Studies on mechanisms and physiopathology of allergic diseases are becoming more and more important because of rapid developments on the concept of disease endotypes and precision medicine. Local allergic rhinitis has been widely considered in the past decade but there is an evolution of concepts [35]. Allergic sensitization at school age is a systemic low-grade inflammatory disorder [36].

Farm exposure protects against development of allergies early in life. High microbial diversity in the environment has been associated with lower asthma risk, particularly in children exposed to farming. A stronger inverse association of asthma with bacterial diversity in mattress dust as compared to nasal samples suggests microbial involvement beyond mere colonization of the upper airways [37]. At 4.5 years, protection against asthma by farm-milk exposure was partially mediated by regulatory T cells (Tregs). However, Tregs at the age of 6 years were decreased with farm exposure and increased within asthmatics, opposite to age 4.5 years. This immunological switch defines a critical ‘time window’ for Treg-mediated asthma protection via environmental exposure before age 6 years [38].

PTTG1IP and MAML3 (pituitary tumour-transforming 1 interacting protein (PTTG1IP) and rs345983 in Mastermind-like 3 (MAML3) are novel genomewide association genes for severity of hyperresponsiveness in adult asthma. The relevance of these genes is supported by co-expression of PTTG1lP with vimentin and E-cadherin-1 [39].

Epithelial barrier dysfunction is a central feature in the pathogenesis of allergic disease. Epithelial-to-mesenchymal transition (EMT) has been proposed as one mechanism afflicting barrier in asthma. A striking suppression of epithelial differentiation was found in asthma, overrepresented by insufficiency in insulin and Notch signaling, but with the absence of conventional EMT markers [40]. EFNB2, FGFR1, FGFR2, INSR, IRS2, NOTCH2, TLE1, and NTRK2 were identified as novel markers central to dysregulation of epithelial-mesenchymal signaling.

Other studies found that an altered fatty acid metabolism and reduced stearoyl-coenzyme a desaturase activity in asthma [41]. Protein phosphatase 5 mediates corticosteroid insensitivity in airway smooth muscle in patients with severe asthma [42].

## Treatment

Efficacy and safety of tiotropium was confirmed in school-age children with moderate-to-severe symptomatic asthma by a systematic review [43].

Although the main symptomatic treatment for rhinoconjunctivitis is intranasal corticosteroids (INCS) (daily or on demand) and oral antihistamines, it remains unclear which treatment provides the best relief of symptoms. A single-blinded randomized controlled trial in children (aged 6–18 years) with pollen-related AR. Patients received either INCS daily (fluticasone propionate), INCS on demand (fluticasone propionate) or oral antihistamine on demand (levocetirizine) for 3 months during the grass pollen season. This trial showed shows that INCS daily was not superior to INCS on demand or to antihistamine on demand regarding the number of symptom-free days. An on-demand INCS strategy has the advantage of a lower overall corticosteroid exposure and less costs [44].

Endotype-driven treatment in chronic upper airway diseases has been delineated in algorithms for allergic rhinitis and chronic rhinosinusitis [45]. This was the basis for the positioning the principles of precision medicine in care pathways for allergic rhinitis and chronic rhinosinusitis—A EUFOREA-ARIA-EPOS-AIRWAYS ICP statement [46].

Lack of adherence in the treatment of asthma or rhinitis is a major problem. Differences in medication adherence are associated with beliefs about medicines in asthma and COPD [47]. There is a role for shared decision-making to reduce the burden of nonadherence among adults with asthma [48]. Serious games in asthma education may help to improve adherence [49]. Mobile Health Intervention can be used in low-income, urban caregivers of children with asthma and may improve adherence [50].

Three papers on omalizumab provided new information. Omalizumab effectively protects against early and late allergic responses in asthma after 4 weeks [51]. Lung function parameters may identify omalizumab responder patients [52]. Galectin-3 is an early predictive biomarker of modulation of airway remodeling in patients with severe asthma treated with omalizumab for 36 months [53].

Alternative treatments were also approached. Lung function improvement and airways inflammation reduction were observed in asthmatic children after a rehabilitation program at moderate altitude [54]. Some guidelines were proposed to treat food allergy with an impact on airway diseases [55, 56].

## Immunotherapy

A large number of studies and meta-analyses were published in 2017. Systematic reviews of allergen immunotherapy were published for allergic asthma and rhinoconjunctivitis [57–60], the prevention of allergy [61], and IgE-mediated food allergy [62]. They were used to provide evidence for the large consensus on immunotherapy carried out by EAACI. The first EAACI guidelines on allergen immunotherapy concerned the prevention of allergy [63]. Efficacy of allergen immunotherapy in reducing the likelihood of developing new allergen sensitizations was questioned in a systematic review [64]. Pooled efficacy and safety data for house dust mite sublingual immunotherapy tablets were studied in adolescents [65].

Clinical benefits and safety were published for the treatment of patients with SQ house dust mite sublingual tablet in house dust mite allergic rhinitis [66], house dust mite sublingual tablet rhinitis [67], sublingual monomeric allergoid tablets in house dust mite-allergic patients [68] and rBet v 1-FV in birch-related soya allergy [69].

The role of immunotherapy in elderly patients should be confirmed. A randomized, double-blind placebo-controlled trial found it was effective [70].

European Survey on Adverse Systemic Reactions in Allergen Immunotherapy (EASSI) represent a real-life clinical assessment in adults [71] and children [72]. 4316 adults were studied. A total of 109 SRs were recorded. Immunotherapy for respiratory allergy is safe, with a low number of SRs observed in real-life clinical practice. A personalized analysis of risk factors could be used to minimize systemic reactions. 1563 children (mean ± SD age: 11.7 ± 3.9 years; rhinitis: 93.7%; asthma: 61.5%; polysensitization: 62.5%) were assessed. 24 patients experienced severe systemic reactions. In a real-life pediatric setting, systemic reactions are infrequent and generally not severe. Pollen polysensitization, grass pollen extracts and natural extracts (vs. allergoids) were risk factors for AIT-associated systemic reactions.

## Costs

The comparison of costs of different treatment approaches has been an unmet need and requires extensive studies. The Canadian Respiratory Research Network studied the 10-year trends in direct costs of asthma. This population-based study on 341,457 individuals (mean age at entry 27.3, 54.1% female) showed that excess costs in patients with asthma were $1028.0 (95% CI $982.7–$1073.4) per patient-year (PY). Medications contributed to the greatest share of excess costs ($471.7/PY). There was a 2.9%/year increase in excess costs, a combination of asthma-attributable costs declining by 0.8%/year while excess costs unrelated to asthma increasing by 3.8%/year. The most dramatic trend was observed in asthma-related outpatient costs, which decreased by %6.6/year [73].

Estimate of the total costs of allergic rhinitis in specialized care in Spain was based on real-world data (FERIN Study) [74]. The total mean cost of AR per patient-year (n = 498) was € 2326.70 (direct, 553.80; indirect, 1772.90). The total cost of AR for society is considerable. A reduction in presenteeism would generate considerable savings for society. Work productivity is largely impaired in days with uncontrolled rhinitis [75]. Costs of perennial allergic rhinitis and allergic asthma increase with severity and poor disease control [76].

In a Portuguese nationwide, population-based, cost-of-illness study, costs of childhood asthma are high (0.9% of the healthcare expenditures in Portugal). Direct costs represented three-fourth of total costs, mainly related to the use of healthcare services for acute asthma care [77].

## Political agenda

Allergic diseases and asthma represent over 25% of the European population and cause a very high burden. Strategies for early diagnosis, prevention and control need to be anchored on a strong political agenda to implement the results of the research into practice. Two important political activities at the EU Parliament were reported in the journals: A European Summit on the Prevention and Self-Management of Chronic Respiratory Diseases (29 March 2017) [78] and a European symposium on the awareness of allergy for the promotional campaign (26–28 April 2016) [79].

## Abbreviations

AR: allergic rhinitis
ARIA: allergic rhinitis and its impact on asthma
AIRWAYS ICPs: integrated care pathways for airway diseases
CHRODIS: adressing chronic diseases and healthy ageing across the life cycle
COPD: chronic obstructive pulmonary disease
EAACI: European Academy of Allergy and Clinical Immunology
EMT: epithelial-to-mesenchymal transition
EPOS: European position paper on rhinosinusitis
EUFOREA: European Forum for Research and Education in Allergy and Airway Diseases
FeNO: exhaled nitric oxide
GA^2^LEN: Global Allergy and Asthma European Network
INCS: intra-nasal corticosteroid
LC-PUFAs: long-chain polyunsaturated fatty acids
miRNAs: MicroRNAs
ST2: IL-33 receptor
